# Phosphaturic mesenchymal tumour of the sinonasal area: case report and review of the literature

**DOI:** 10.1186/1758-3284-3-16

**Published:** 2011-03-16

**Authors:** Pavel Komínek, Ivo Stárek, Marie Geierová, Petr Matoušek, Karol Zeleník

**Affiliations:** 1 Department of Otorhinolaryngology, University Hospital Ostrava, Czech Republic; 2 Department of Otorhinolaryngology, Palacký University Olomouc, Czech Republic; 3 Department of Pathology, Faculty Hospital, Palacký University Olomouc, Czech Republic

**Keywords:** phosphaturic tumour, oncogenous osteomalacia

## Abstract

**Background:**

Oncogenous osteomalacia (OOM), which is also known as tumour-induced osteomalacia, is a rare condition associated with a neoplasm and a related systemic bone demineralization caused by renal phosphate wasting. OOM usually occurs in association with a variety of different mesenchymal tumours, and they were categorized into four distinct morphological patterns which they termed "phosphaturic mesenchymal tumour". Of its 4 histopathological subtypes, the mixed connective tissue variant is most commonly observed. Only 10% of cases appear in the head and neck regions and moreover, only 5 previously published tumors were localized in the sinonasal area. The authors describe a case of a man with a PMT originating from the frontoethmoidal region.

**Case presentation:**

A 53-year-old man was referred to our ORL clinic due to a presence of a mass at the nasal root having been growing asymptomatically for 1 year. CT scans demonstrated a large (25 × 20 × 35 mm) bilateral frontoethmoidal mass with destruction of nasal bones. The tumor did not appear to invade to the anterior skull base. A selective angiography revealed a moderate hypervascularization of the tumour during early and late arterial phases. The tumour was removed from the external approach and the definitive histopathological diagnosis was a phospaturic mesenchymal tumor. Dual energy X-ray absorptiometry revealed a slight osteopenia of the first and second lumbar vertebrae and neck of the thigh bone. The serum and urinary levels of both calcium and anorganic phosphate were within normal limits. The patient is doing well three years after the operation, and the serum and urine levels of calcium and phosphate remain well within normal limits.

**Conclusion:**

PMT is rare in the sinonasal region, it can be rarely observed without the signs of osteomalacia.

## Introduction

Oncogenous osteomalacia (OOM), which is also known as tumour-induced osteomalacia, is a rare condition associated with a neoplasm and a related systemic bone demineralization caused by renal phosphate wasting [1,2]. In 1947, unaware of the causative relation between phosphate wasting and this neoplasm, McCance published the first case [1]. OOM usually occurs in association with a variety of different tumour types, frequently very small, a fact which makes their discovery difficult [2-4]. Most published cases were presented by various soft tissue, bone neoplasms, and pseudotumors [4,5]. In 1987 Weidner and Santa Cruz revealed that many of these mesenchymal tumours were histologically polymorphous, and they were categorized into four distinct morphological patterns which they termed "phosphaturic mesenchymal tumour" (PMT), comprising four subtypes [4]. The most common one was a mixed connective tissue variant (MCT), composed of primitive mesenchymal cells. Quite recently, Folpe and colleagues have reviewed 32 personal and 109 reported mesenchymal OO-associated tumors, the latter representing the majority of all published (English literature) cases [5]. 102 out of the total of 141 various mesenchymal tumors were reclassified as true or probable phosphaturic mesenchymal tumor mixed connective tissue variant (PMTMCT) and the above-mentioned three other variants of PMT. Only 5 previously published tumors were localized in the sinonasal area [5].

Because of its scarcity, most ENT surgeons remain oblivious to the existence of PMTMCT. Here, the case of a 53-year-old man with a PMTMCT, originating bilaterally from the frontoethmoidal region, is described.

## Case Report

**Figure 1:**
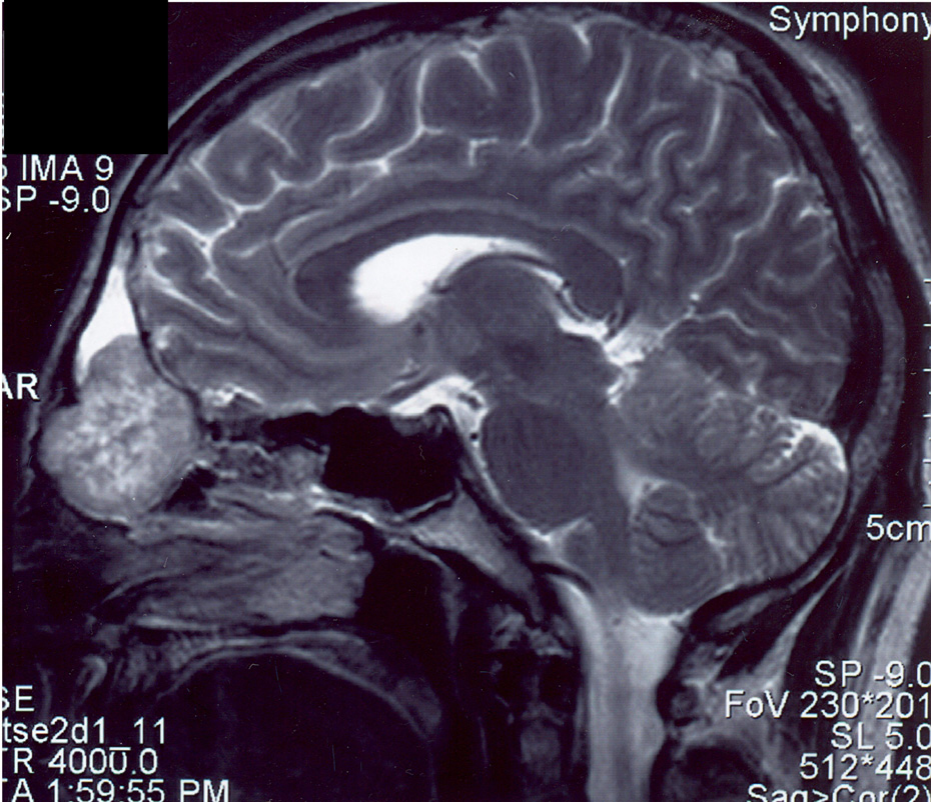
**T2W MRI sagittal scan**. Well bordered tumorous masses of the nasal cavity, heterogeneous, and more hyperintensive structures, destruction of the surrounding skeleton, prominence of the masses into the bases of frontal sinuses, the rest filled with liquid. No intra-cranial growth - lamina interna of the frontal sinus preserved - narrow hem with no signal.

**Figure 2:**
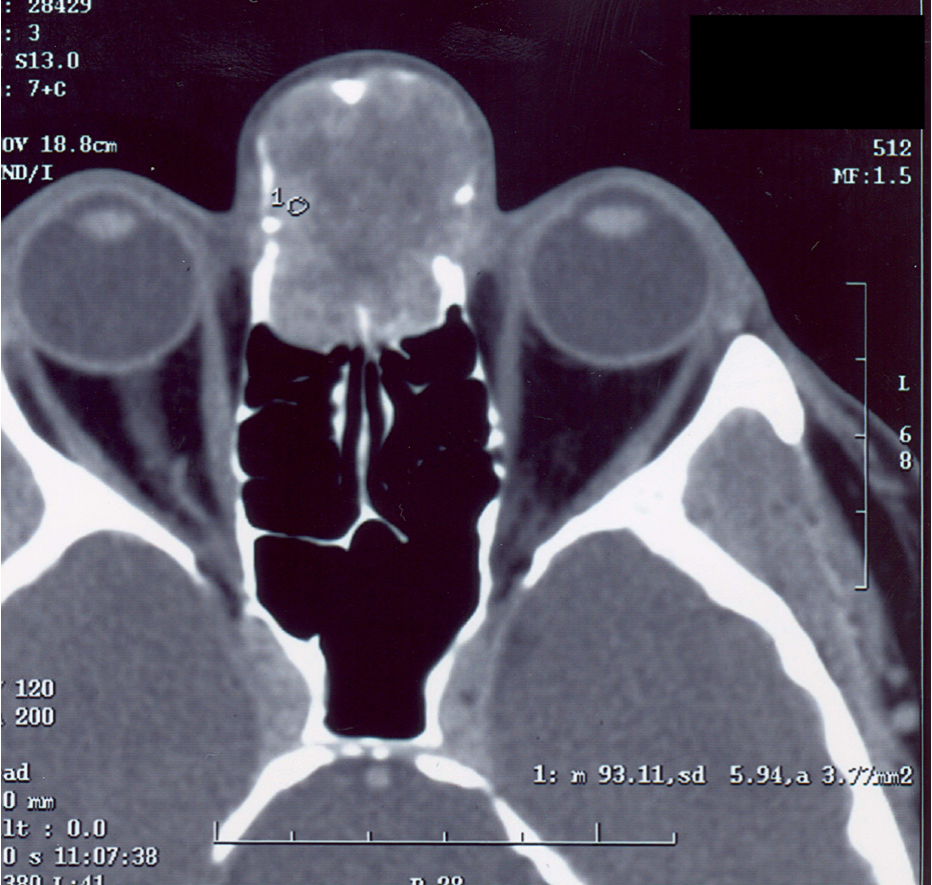
**Axial CT scan**. Well bordered soft-tissue tumormasses of a middle signal intensity with destruction of the nasal bones, usuration or even destruction of the medial orbital wall. Broadened nose root, prominence of the masses dorsally, up to the frontal ethmoid sinuses, rear ethmoides and sphenoidal sinus of normalairy character.

A 53-year-old man was referred to our ORL clinic due to a presence of a mass at the nasal root having been growing asymptomatically for 1 year. The patient's past medical history was unremarkable. The root of the nose was enlarged by a non-tender, firm, ovoid mass, the overlaying skin was intact. Nasal endoscopy revealed a smooth bulge in the superior turbinate apparent on both sides. An endoscopic biopsy was performed, and was accompanied by profuse bleeding. The tentative histopathological diagnosis was a juvenile angiofibroma. A computed tomography (CT) scan demonstrated a large (25 × 20 × 35 mm) bilateral frontoethmoidal, strongly enhancing (90 HU) mass with concomitant destruction of nasal bones, the frontal processes of the maxilla, inferior wall of the frontal sinuses and medial orbital wall. MRI scans demonstrated that the tumour did not appear to invade the anterior skull base [Figure 1, 2]. A selective bilateral carotid angiography revealed a moderate hypervascularization (tortuous arteries) of the tumour during early and late arterial phases. The hypervascular area was supplied from internal and external carotid arteries through the ophthalmic and maxillary arteries. In the parenchymatous phase, a moderate tumour blush was visible. Dual energy X-ray absorptiometry revealed a slight osteopenia of the first and second lumbar vertebrae and neck of the thigh bone. The serum and urinary levels of both calcium and anorganic phosphate were within normal limits. Neither the clinical picture nor all the above-mentioned findings were consistent with osteomalacia.

**Figure 3:**
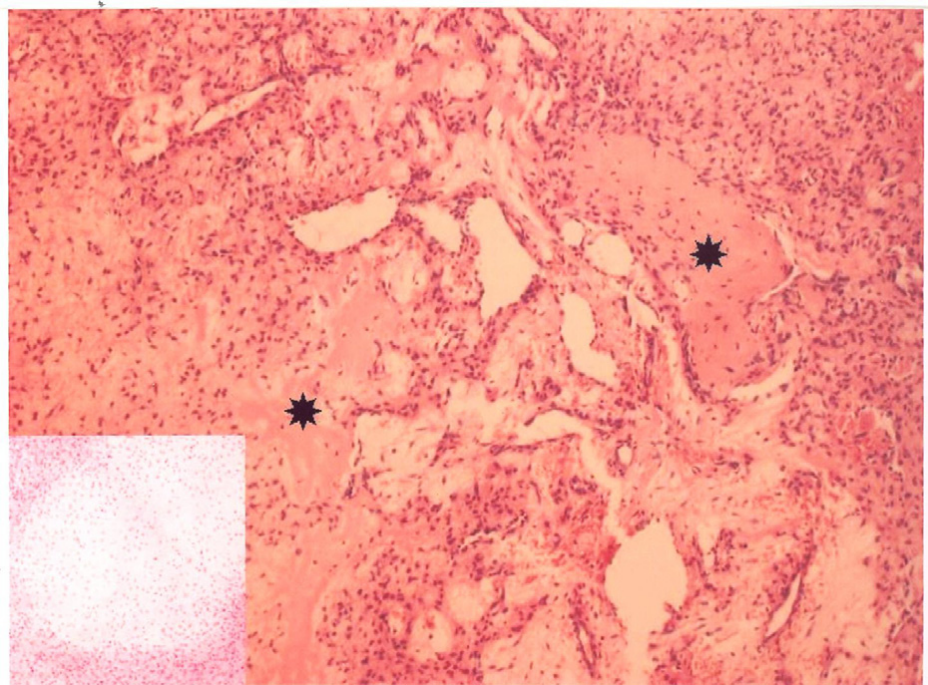
**A photomicrograph showing numerous small mesenchymal cells, vascular channels, and osteoid areas (asterisk) (H & E, 10×)**. Inset: chondroid differentiation of the stroma (H & E, 200×)

The tumour was removed under general anesthesia. After an H-shaped skin incision was made, we encountered a grayish-white mass invested in a thin capsule. The tumour was easily extirpated from the intact posterior wall of the frontal sinuses, anterior ethmoids, and nasal cavity, and the posterosuperior part of the nasal septum was also removed. The peroperative bleeding was moderate, and it ceased immediately after the tumour resection. The definitive histopathological diagnosis was a PMTMCT variant [Figure 3]. It consisted of benign (in appearance) undifferentiated mesenchymal spindle- to stellate-shaped cells. The cells were negative for CD 34, CD 99, S-100, AE1 and AE3. The examination for smooth-muscle actin was focally positive, a strong immunoreactivity for vimentin was noted. Osteoclast-like giant cells were not found. In some parts of the tumour a chondroid and osteoid differentiation was noticed, as was a moderate microvasculature comprised of capillary-sized vessels invested in endothelial cells. Some vessels showed a staghorn branching pattern. No mitoses, atypia, foci of necrosis or bleeding were observed in the tumour during the histology assessment.

The course, thereafter, was uneventful, and two years after the operation the patient is doing well; there is no evidence of the disease in either endoscopic or CT examinations, and the serum and urine levels of calcium and phosphate remain well within normal limits.

## Discussion

Phosphaturic mesenchymal tumour (PMT) is representative of a very rare group of neoplasms, usually benign [2,5,6]. PMT with the mixed connective tissue variant (PMTMCT) is the most commonly observed, while the remaining minority consists of the three other histopathological subtypes (an osteoblastoma-like tumour, a non-ossifying fibroma-like tumour and an ossifying fibroma-like tumour) [3,4]. They are distinguished from other mesenchymal tumours by the expression of a number of gene products, which are related to bone matrix formation, mineralisation and mineral ion transport [3,7].

These tumours may occur in almost any location; sinonasal area is affected very rarely [1,2,5,7]. In the retrospective analysis of 109 cases reported in the English language literature, Folpe found only 12 PMTMCTs and 1 ossifying fibroma-like PMT (13%) localized in the head and neck region [5]. Five of these 13 tumours developed in the sinonasal area (Table 1) [6,8-10].

**Table 1:** A review of sinonasal PMT MCT

Author	Sex, age	Location	Therapy	Follow-up interval/tumour/signs of OO
Koriyama [2]	F, 41y	max. sinus	surgical	not indicated

Weidner [4]	F, 39 y	max. sinus, infratemporal fossa invasion, right	surgical removal	27 mo/recurrence/present

Gonzales [6]	F, 69 y	max. and frontal sinus, ethmoids, intracranial invasion, right	none	died of tumor-related reasons

Linsey [8]	F, 54 y	nasopharynx	surgical removal	not indicated

Papotti [9]	F, 38 y	max. sinus, ethmoids, nasal cavity, orbital floor invasion, left	radiotherapy part. surg. removal	18 mo/not indicated/present

Kawai [10]	F, 53 y	nasal cavity, ethmoids, right	surgical removal	not indicated

present case	M, 53 y	frontal sinus, ethmoids, nasal cavity billat	surgical removal	24 mo/no evidence of tumor/primarily not present

OO - oncogenous osteomalacia

mo - months

F - female

M - male

The mechanism of the tumour-induced osteomalacia remained unclear for many years but all the evidence pointed to a circulating phosphaturic agent [2,3,7,11]. Recently, it has been demonstrated that some OOM-associated tumours, including PMTMCT, over express fibroblast growth factor FGF-23, a protein, which inhibits renal phosphate reabsorption by a mechanism distinct from that of other known phosphate homeostasis hormones [2,3,11]. The precise role of FGF-23 in the pathogenesis of OOM is uncertain, but most FGFs are potent stimulators of angiogenesis *in vitro *and *in vivo *[3].

There were neither clinical nor laboratory signs of osteomalacia in our patient with the tumour, which met unambiguously all histopathological criteria for a PMTMCT. Similarly, Folpe *et al *in their study revealed 3 cases of PMCMCT without a known history of phosphaturia, which they had considered a non-phosphaturic variant of this histopathologic entity [5]. They speculated that in such cases the tumour either secreted inactive or insufficient FGF-23, or even none whatsoever. Another possible explanation could be the patient's capacity to compensate for the increased secretion of this factor in another manner [5]. Unfortunately, since the FGF-23 antibody is not commercially available, we failed to test the above-described tumour for that factor. In this respect, our case was not contributory. For establishing the histopathological diagnosis of a PMTMCT, FGF-23 immunohistochemistry is not crucial.

PMTMCT is basically a benign lesion, histologically malignant, metastasizing variants of which happen to occur extremely rarely [5,7]. Nonetheless, infiltration and invasion of surrounding tissues are very frequent features of this otherwise benign-appearing PMTMCT [3,5].

In general, a reasonable treatment protocol for PMT MTC is a complete surgical removal, which dramatically resolves the tumour-associated osteomalacia (known for its resistance to conservative therapy). Owing to its local invasiveness, the lesion should be removed using wide margins of resection [2,5-7]. Remnants of the tumour may be inadvertently left behind, threatening the patient with serious local and systemic complications resulting from continuous growth and renal phosphate wasting, respectively [5]. A postoperative laboratory and radiological follow-up is thus necessary [2].

## Conclusion

In most patients with oncogenous osteomalacia, the causative tumour is a PMTMCT. About 5% of all these lesions originate in the frontoethmoidal area. Here, the tumour may easily go unrecognized until a thorough battery of CT scans is performed. Local invasion is a characteristic feature of benign PMTMCT, requiring wide-margin resection. Given the spatial limitations imposed by surrounding anatomical structures, residual tumour may be left behind in that area, making careful follow-ups an absolute necessity.

## Competing interests

There is no type of financial interest that is related to the manuscrip, including stock or ownership of a business entity connected to a product described in the paper, paid consulting for the company or competing companies, or patent rights to a drug or piece of equipment.

## Authors' contributions

PK performed the operation and is the main author and supervisor of the manuscript.

IS performed preoperative diagnostics, did follow-up, drafted the manuscript, cooperated during the manuscript preparing,.

MG carried out histological analysis and wrote histological part of the manuscript.

PM did searching and analysis of the literature, prepared the table and participated in the design of the paper.

KZ was the opponent of the manuscript, did critical revision of the manuscript and participated on the design of the paper, was responsible for the picture preparing.

All authors read and approved the final manuscript.
